# Bayesian Spatio-Temporal Prediction and Counterfactual Generation: An Application in Non-Pharmaceutical Interventions in COVID-19

**DOI:** 10.3390/v15020325

**Published:** 2023-01-24

**Authors:** Andrew Lawson, Chawarat Rotejanaprasert

**Affiliations:** 1 Department of Public Health Sciences, College of Medicine, Medical University of South Carolina, Charleston, SC 29466, USA; 2 Usher Institute, School of Medicine, University of Edinburgh, Edinburgh EH16 4TJ, UK; 3 Department of Tropical Hygiene, Faculty of Tropical Medicine, Mahidol University, Bangkok 10400, Thailand; 4 Mahidol-Oxford Tropical Medicine Research Unit, Faculty of Tropical Medicine, Mahidol University, Bangkok 10400, Thailand

**Keywords:** Bayesian, counterfactual, causal, nowcasting, spatio-temporal, prediction, non-pharmaceutical interventions, NPIs

## Abstract

The spatio-temporal course of an epidemic (such as COVID-19) can be significantly affected by non-pharmaceutical interventions (NPIs) such as full or partial lockdowns. Bayesian Susceptible-Infected-Removed (SIR) models can be applied to the spatio-temporal spread of infectious diseases (STIFs) (such as COVID-19). In causal inference, it is classically of interest to investigate the counterfactuals. In the context of STIF, it is possible to use nowcasting to assess the possible counterfactual realization of disease in an incidence that would have been evidenced with no NPI. Classic lagged dependency spatio-temporal IF models are discussed, and the importance of the ST component in nowcasting is assessed. Real examples of lockdowns for COVID-19 in two US states during 2020 and 2021 are provided. The degeneracy in prediction over longer time periods is highlighted, and the wide confidence intervals characterize the forecasts. For SC, the early and short lockdown contrasted with the longer NJ intervention. The approach here demonstrated marked differences in spatio-temporal disparities across counties with respect to an adherence to counterfactual predictions.

## 1. Introduction

During the COVID-19 pandemic period of 2020, many countries worldwide enacted lockdowns to try to control the spread of the virus. These lockdowns are examples of non-pharmaceutical interventions (NPIs), and they were used mainly prior to the availability of vaccination.

The CDC notes that:

Nonpharmaceutical Interventions (NPIs) are actions, apart from getting vaccinated and taking medicine, that people and communities can take to help slow the spread of illnesses such as pandemic influenza (flu). NPIs are also known as community mitigation strategies. When a new flu virus spreads among people, causing illness worldwide, it is called a pandemic flu. Because a pandemic flu virus is new, the human population has little or no immunity against it. This allows the virus to spread quickly from person to person worldwide. NPIs are among the best ways of controlling pandemic flu when vaccines are not yet available.

(https://www.cdc.gov/nonpharmaceutical-interventions/ (accessed on 20 January 2023))

NPIs can take various forms, and they can extend for different time periods. In the US, many southern states enacted lockdowns for only a few weeks, whereas northern state lockdowns were longer. In some cases, only partial lockdowns were observed, whereby some businesses remained open, but e.g., schools were shut. In the US state of South Carolina (SC), initial case reports in early March 2020, followed by the pandemic declaration by WHO (12 March), led to a state of emergency declaration on 13 March and school closures on 15 March; restaurants closed on 17 March, and on 19 March, non-essential state employees and colleges were to shelter in place.

Not until 1 April did the state authorize the closure of non-essential businesses. 3 April saw the introduction of travel restrictions, and on 7 April, a full lockdown with non-essential travel banned and work at home was ordered. 

By 21 April, retail stores were allowed to reopen, and by 4 May, the home and work order was lifted, and outdoor dining was allowed. Finally, by 11 June, all restrictions were lifted. In effect, the main full lockdown lasted only 2 weeks. 

**Figure 1:**
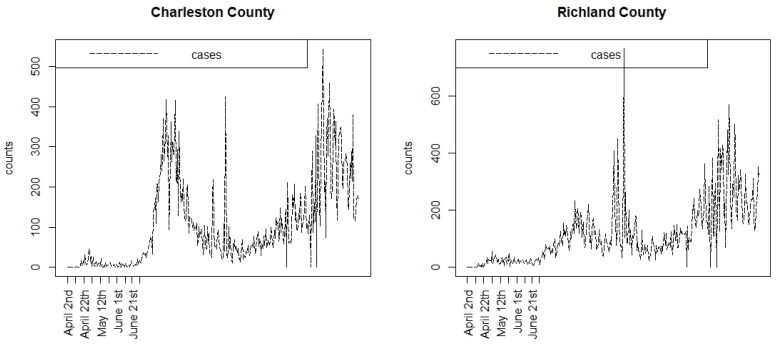
Case count profiles for two South Carolina counties during the first 353 days of the pandemic. Only the early lockdown dates are shown.

Figure 1 displays the case count time profiles for the Charleston and Richland counties in SC during the first part of the pandemic, for 353 days up to the end of February 2021. Listed are the early dates related to the lockdowns in 2020. It is notable that following the full lifting of lockdowns in June 2020, there were significant increases in the case counts leading into the large summer wave. Whether the partial or full lockdowns were effective in controlling the early spread is difficult to ascertain. 

## 2. Assessing the Effects of NP Interventions 

It is clear that NPIs have to be compared to situations where interventions have not been introduced. This leads to a difficulty in that finding a suitably matched location or time period with null conditions which can be used as a comparator is crucial. With time series, it is possible forecast future outcomes based on currently observed data. As an extension to this, it is sometimes useful to make predictions based on lagged observations when the current data or recent data are lacking. This prediction is termed nowcasting [1,2]. It has been applied extensively in economic research, and is now being adopted in infectious disease epidemiology for making health outcome predictions [3]. More recently, during the COVID-19 pandemic, the use of nowcasting has been proposed to generate predictions for the modifications of social mobility during NPIs [4]. An area that has not been examined is the use of nowcasting to make counterfactual predictions of health outcome events. In particular, the use of observed case count data to predict case counts which are altered by NPIs could be a useful approach in understanding the effects of such interventions.

The aim of this paper is to demonstrate the use of nowcasting with Bayesian spatio-temporal models in application to the evaluation of the performance of lockdown NPIs at the county level in two contrasting states in the US: South Carolina (SC) and New Jersey (NJ). Our choice of state to examine is based on the contrast between the population structure and the political structure of the respective states during the pandemic. SC is a southern state which had a Republican governor and a small mainly rural or semi-rural population (5.2 million), whereas NJ is a northern state with a Democrat governor and a large highly urban population (8.88 million). In each state, different NPIs were adopted, and it is our aim to ascertain how effective these were. Our focus is on the case count data only, and we do not examine the mortality counterfactuals, although these could also be a focus.

In the next section, we outline the models evaluated in this study, the generation of counterfactuals, and their comparative evaluations using differential metrics. The data used were made available from the NYT GitHub repository (https://github.com/nytimes/covid-19-data, accessed on 29 November 2022), which has recorded cases and death counts from the State Departments of Health (cases) and the National Center for Health Statistics (NCHS) (deaths) during the course of the pandemic. 

The data used here are in the form of daily case and death counts for each county in each state for the period of 353 days from 6 March 2020 to 21 February 2021. Death counts are used only for updating the susceptible population within the case count models, and they themselves are not modeled.

## 3. The Bayesian Spatiotemporal Case Model

A number of approaches could be considered for the modeling of case count data of the above kind. For example, conventional mathematical models could be used [5], although these do not usually provide statistical error estimates, nor spatial referencing. Our spatial SIR models are essentially extensions of the difference representations of these models embedded within a statistical modeling framework. Time series or machine learning models could be employed to model counts within separate regions (see e.g., [6,7]). While these approaches can yield flexible results, they do not address the spatial structure of the epidemic spread. The pandemic clearly crosses boundaries with personal mobility [8]. In addition, our Bayesian SIR models address the data quality directly (daily count data), and they allow for error estimation from count data models and the appropriate confidence interval estimation. 

Lawson and Kim (2021) [9] proposed a Bayesian spatio-temporal COVID-19 case count model, and this was evaluated on the first 88 days of the pandemic in the counties of SC. Subsequently, this model was extended and updated for the analysis of 353 days [10]. The later analysis of the three waves included a wide range of potential models and modeling strategies. Our models for counterfactuals are based on the retrospective analysis results found.

We define the case count as $y_{ij}$ in the *i* th area and in the *j* th time period. In our example, the areas are counties and the time period is days. For SC, the number of counties is m=46, and for NJ, it is m=22. The total time period is T = 353 days. 

As the spread of infection is an important component of infectious disease modeling, we assume a Susceptible-Infected-Removed (SIR) model for the process. Essentially,

(1) $$\begin{array}{l} y_{ij}∼Pois(\mu_{ij})\\ \mu_{ij}=S_{ij}.\exp(p_{i,j-1}^{r}) \end{array}$$

where $S_{ij}$ is the susceptible population in the *i*,*j* th unit and $p_{i,j-1}^{r}$ is a propagator that allows for the transmission as a function of previous counts and related factors. 

An example of a simple propagator could be

(2) $$p_{i,j-1}^{r}=\alpha_{0}+\alpha_{1}log(y_{i,j-1})+v_{i}$$

where there is a constant intercept acting as a log transmission rate, a dependence on the previous infection count in the given county, and a final random effect term $v_{i}$ which allows for extra variation.

Different specifications of $p_{i,j-1}^{r}$ lead to a range of possible models. In these models, the susceptible pool evolves over time, based on an accounting Equation:

(3) $$S_{i,j}=S_{i,j-1}-I_{i,j-1}-R_{i,j-1}$$

where $I_{i,j-1}$is the true infective count at the previous time, which is a function of $y_{i,j-1}$.

The relationship between the true infective count and the observed count depends on the level of undetected cases. This could be related to the testing frequency, and also to unobserved asymptomatic transmission. Previous studies have noted a variety of asymptomatic rates during the pandemic (e.g., [11,12]). We assumed a rate of 20%, which is a reasonable compromise between the previous levels reported for different population groups. ^12^ Hence, we assume that the true infective count is a scaled version of the observed count: $I_{i,j-1}=\lambda y_{i,j-1}$. The removal term can also be specified as a function of the infective numbers. It is also a function of mortality, and so the total removal can be specified as

(4) $$R_{i,j}=\gamma I_{i,j}+d_{i,j}$$

where $d_{i,j}$ is the current death count. The scaling parameter (γ) can be fixed. In this case, it was assumed to be 0.1. However, a range of values has been examined for this parameter, and the resulting analysis was not affected by this choice.

In previous work [10], it was found that, out of a range of potential models, for South Carolina counties, the model with propagator

(5) $$p_{i,j-1}^{r}=\alpha_{0}+\alpha_{1}log(y_{i,j-1})+\alpha_{2}\log(\sum_{k\in \delta_{i}}y_{k,j-1})+v_{i}+x_{i}^{t}\beta \;\;(\mathrm{SC}1)$$

had the lowest WAIC. In this model, the $\sum_{k\in \delta_{i}}y_{k,j-1}$ term represents a neighborhood effect (the sum of previous count over the neighborhood set $\delta_{i}$, while $x_{i}^{t}\beta$ is a linear predictor involving county-level SES predictors (% under the poverty line, % black population, multidimensional deprivation index for 2017 (https://www.census.gov/library/publications/2019/acs/acs-40.html, accessed on 29 November 2022). In the case of New Jersey, a similar modeling strategy led to the choice of the propagator

(6) $$p_{i,j-1}^{r}=\alpha_{0}+\alpha_{1}log(y_{i,j-1})+\alpha_{2}\log(\sum_{k\in \delta_{i}}y_{k,j-1})+v_{i}+u_{i}+x_{i}^{t}\beta \;(\mathrm{NJ}1)$$

where the term $u_{i}$ is a spatially correlated effect, and $x_{i}^{t}\beta$ is a linear predictor, as above. The spatially correlated term was assumed to follow an ICAR prior distribution [13], and the uncorrelated effect $v_{i}$ has a zero mean Gaussian distribution:

(7) $$\begin{array}{l} v_{i}∼N(0,\tau_{v}^{-1})\\ u_{i}|\{u_{k}{\}}_{k\neq i}∼N({\bar{u}}_{\delta_{i}},\tau_{u}^{-1}/n_{\delta_{i}}) \end{array}$$

where ${\bar{u}}_{\delta_{i}}$ is the mean of u in the neighborhood of the *i* th county. The model with this $u_{i}$ term was not selected in the SC example, which suggests that there is more heterogeneity present in the NJ case.

## 4. Death Count Modeling 

Death counts are also observed, and these are usually related to case numbers, either current or lagged. It is unlikely that deaths for COVID-19 could arise without there being a case reported (at least in the main epidemic period), and so the dependence on lagged case counts is a reasonable assumption. The current death count is defined to be $d_{i,j}$, and once again, we assume a Poisson data model, so that $d_{i,j}∼Pois(\mu_{i,j}^{d})$. Here, the mean death count is parameterized as

(8) $$\begin{array}{l} \log(\mu_{ij}^{d})=\alpha_{0}^{d}+\alpha_{1j}^{d}\log(y_{i,j})+\alpha_{2j}^{d}\log(T_{i,j-1})+v_{i}^{d}\;\;\;\;\mathrm{DC}1\\ where\;T_{ij}=\sum_{k=1:j}y_{i,k}. \end{array}$$

The form of the dependence relies on the need to make the deaths dependent on counts, but with a potential lag of undefined length. Hence, it is assumed that cumulative case counts should be included, as well as the current case number. This model form has been found to provide a good fit to mortality data in the pandemic [10,14].

## 5. Nowcasting and Counterfactuals 

Nowcasting is often used in situations where infectious disease is being monitored but where a reporting delay occurs [3,14,15]. This delay can lead to a bias such as under-reporting or mis-attribution. To alleviate this delay bias, a form of forecasting is used, whereby the projections of case numbers are made from the existing data up to the current time. Once the updated data are available, then the count is adjusted. The process is continued until the final time point of the study. 

This form of missing data forecasting can be applied in other situations. Non-pharmaceutical interventions (NPIs) are often implemented during epidemic periods to try to reduce the spread of disease. These interventions often require spatial restrictions, such as social distancing and mobility constraints such as travel/work bans, or ‘work at home’ mandates and business closures. These are often referred to as lockdowns. During the early part of 2020, many places around the globe implemented lockdowns of various forms to reduce COVID-19 spread. These usually took the form of gradual business and school closures, and final travel bans. 

In this paper we examine the use of nowcasting to try to predict the effects of lockdown, or their lifting, on the COVID-19 experience in two contrasting US states: South Carolina (SC) and New Jersey (NJ). SC is a southern state with a small population (~5 million) and only small urban centers (Charleston, Columbia, Greenville, and Spartanburg). NJ is an urbanized state with a much larger population (~9 million), and it has (partly) suburban population centers of Trenton, Newark, Jersey City, and Atlantic City bordering the city of New York. We examine the county level case counts of COVID-19 during the lockdown periods relevant to SC and NJ. These periods differ, as the state governors decided to implement different types and periods of lockdown. For SC, the lockdown started on 13 March and the partial lifting of lockdown happened on 31 March (18 days). The final lifting occurred on 13 May, but many activities were resumed before this date. For NJ, the lockdown was prolonged until 9 June (80 days) following a partial lockdown from 9 March until 21 March. The use of a counterfactual generation for the COVID-19 NPIs was proposed for employment data previously [4]. The application of the counterfactual generation to spatio-temporal COVID-19 modeling has not been reported before.

## 6. Counterfactual Generation 

Consider the historical case count data, and assume that a good model is known for these data. We will return to the definition of a good or ‘best’ model at a later stage. For that good model at a fixed time (T), a prediction from the model is made. For a spatio-temporal model, this prediction is made for all regions under study: in this case, counties. Unsupervised prediction for K time units is used to assess what the effect of continuation under a pre-T model has compared to the actual observed count over the K time periods. The differences between the observed and counterfactual (predicted) count are then summarized, and a comparison is made between the SC and NJ state level responses.

The algorithm steps are:

(1) Retrospectively fit the ‘best’ model for data, up to and including time T.
(2) Essentially, we use MCMC sampling from the converged posterior up to T. A large parameter sample is then taken, and the SIR count model is allowed to evolve to time T + K, so that a set of predicted counts $y_{i,T+1}^{p}........y_{i,T+K}^{p}$ is generated using

(9) {yi,kp}~Pois({Si,k.exp(pi,k)}){pi,k}={α0}+{α1}log(yi,k−1p)+{α2}log(∑l∈δiyl,k−1p)+{vi}+xit{β}{} denotes the sampled parameter set δi is the neighborhood set of the i th region

This is essentially generating predictions from SC1. For NJ1, an added ICAR term is included. Note that death counts must also be generated, as the case predictions will be a function of the accounting equation, which is a function of the concurrent death count. These are generated from the ‘best’ death count model. In this case, it is assumed to be DC1. 

In this way, a counterfactual is generated in each county and for each time period, which can then be compared with the observed count during the NPI. For SC, the best model used was that found during a retrospective model search of a wide range of potential models (SC1). A similar search for NJ models led to the use of NJ1 as the ‘best model’ [10].

All Bayesian models were fitted using posterior sampling, based on the R package Nimble [16] This allows for the specification of a range of likelihood and prior models, and the sampling of the posterior functionals generated using the above algorithm. 

## 7. South Carolina Counties

We assumed that the crucial time points for this state, measured from the first case, 6 March, were T = { 26,42,68}. The first marks the initiation of lockdown; the second, the partial lifting; and third is the final lifting of the lockdown (13 May). Examined were counterfactuals of lengths 16, 26, and 40. The final end date was 22 June. 

**Figure 2:**
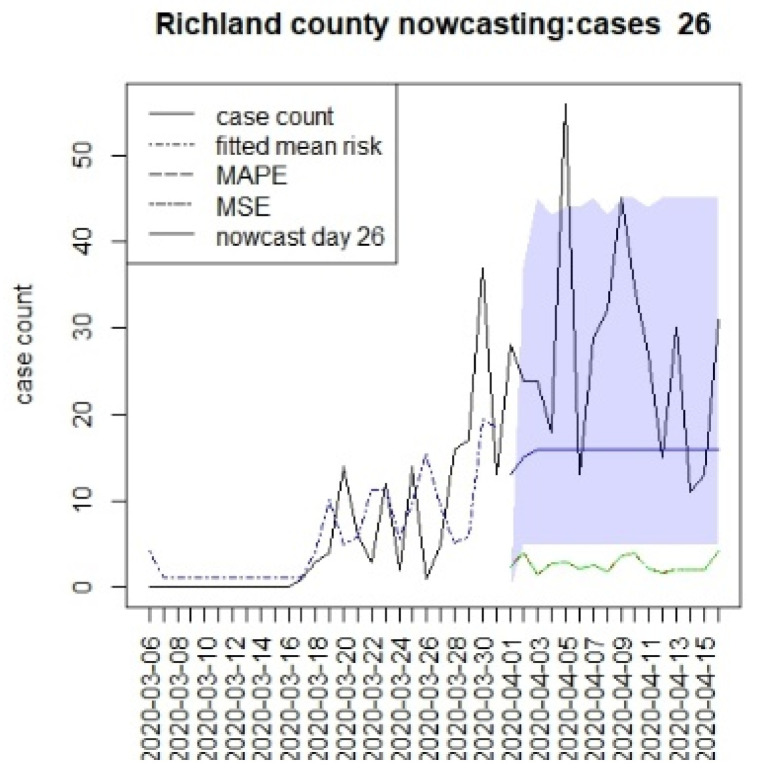
Counterfactual for Richland county at T = 26.

**Figure 3:**
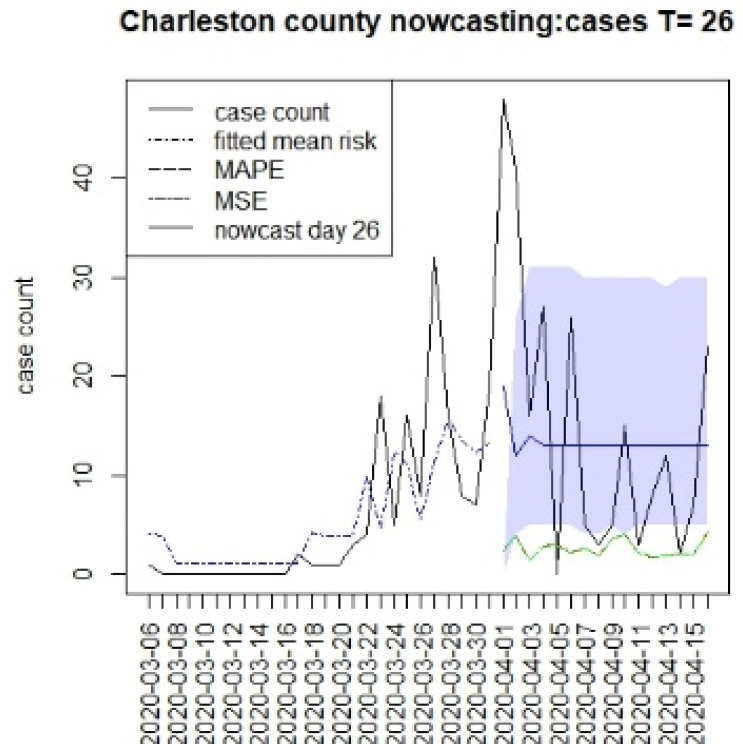
Counterfactual for Charleston county at T = 26.

**Figure 4:**
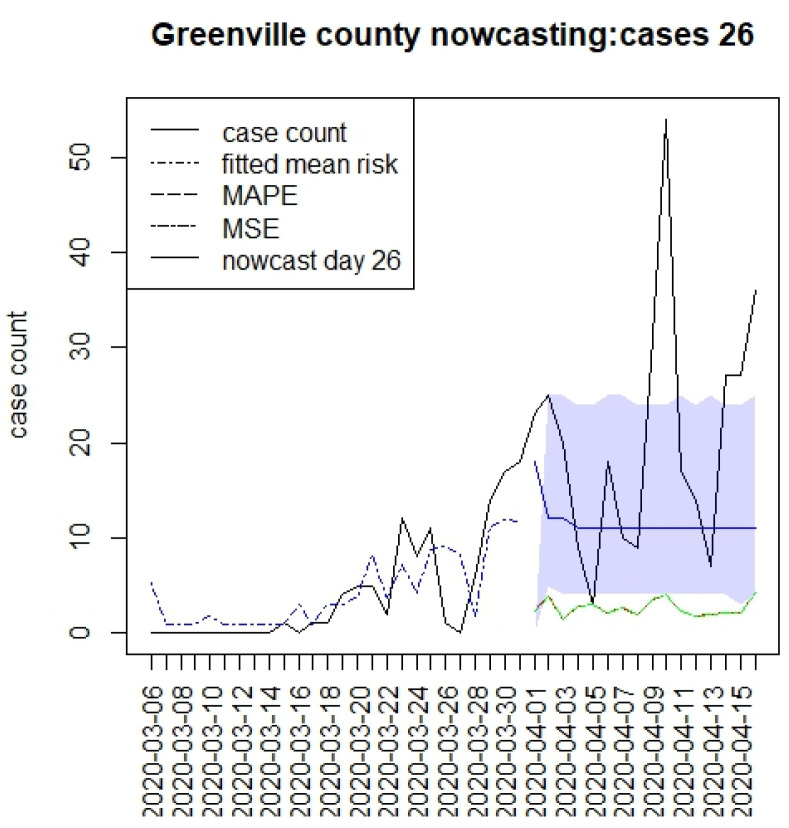
Counterfactual for Greenville county at T = 26.

**Figure 5:**
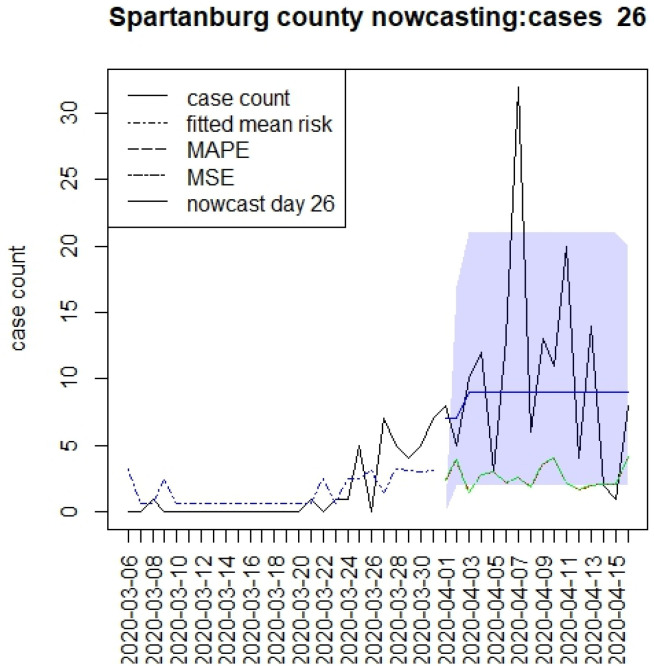
Counterfactual for Spartanburg county at T = 26.

Figure 2, Figure 3, Figure 4 and Figure 5 display the results for four SC counites at T = 26. In these displays, the counterfactual is denoted by a thin solid purple line. The 95% credible interval for the counterfactual is shown in purple shading. The mean squared error of the model fit and mean absolute predictive error is also shown. It is notable that for this first period until mid-April, Richland is below the observed count, and Charleston is mostly higher than that observed. Greenville and Spartanburg show a variable picture with many spikes of cases, followed by gaps during this period. 

**Figure 6:**
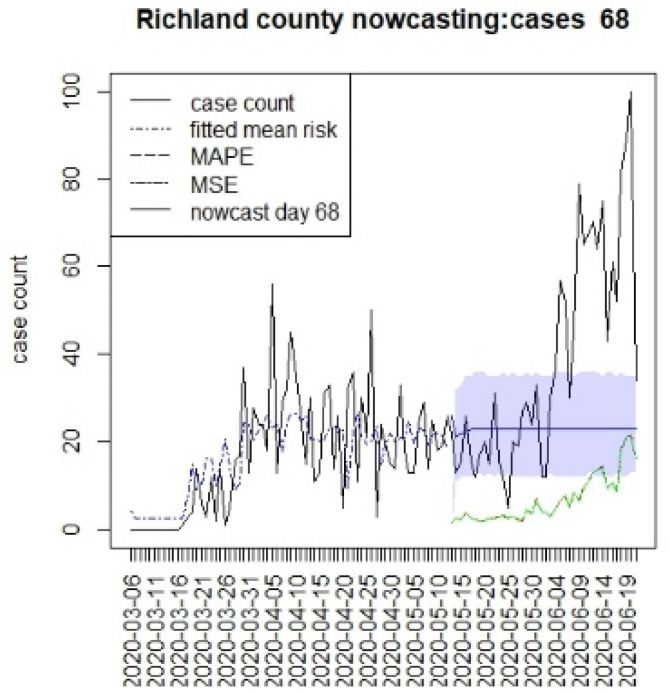
Counterfactual for Richland county at T = 68.

**Figure 7:**
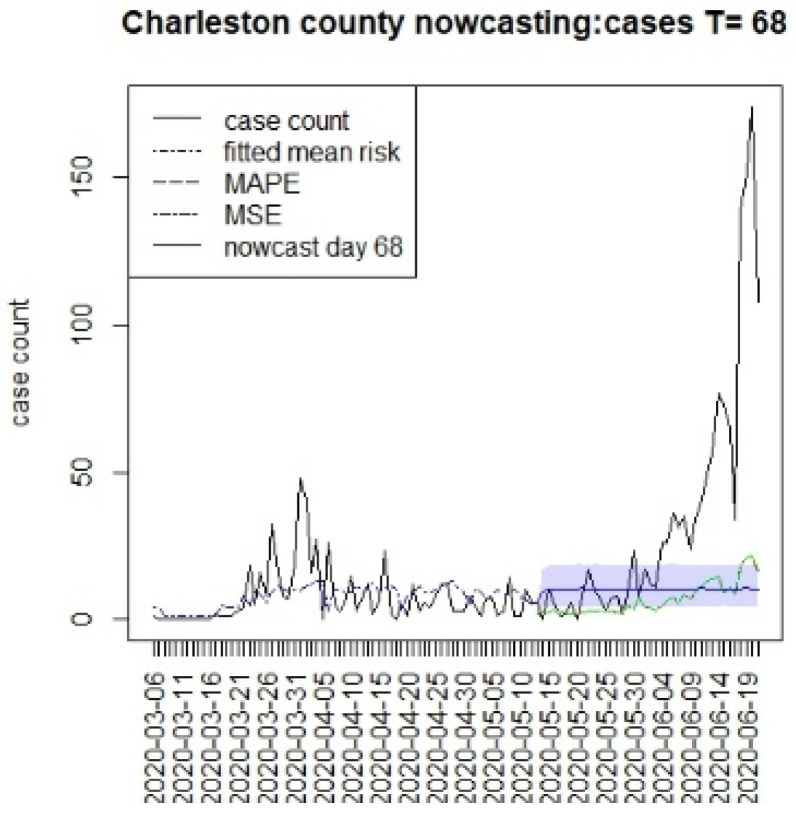
Counterfactual for Charleston county at T = 68.

**Figure 8:**
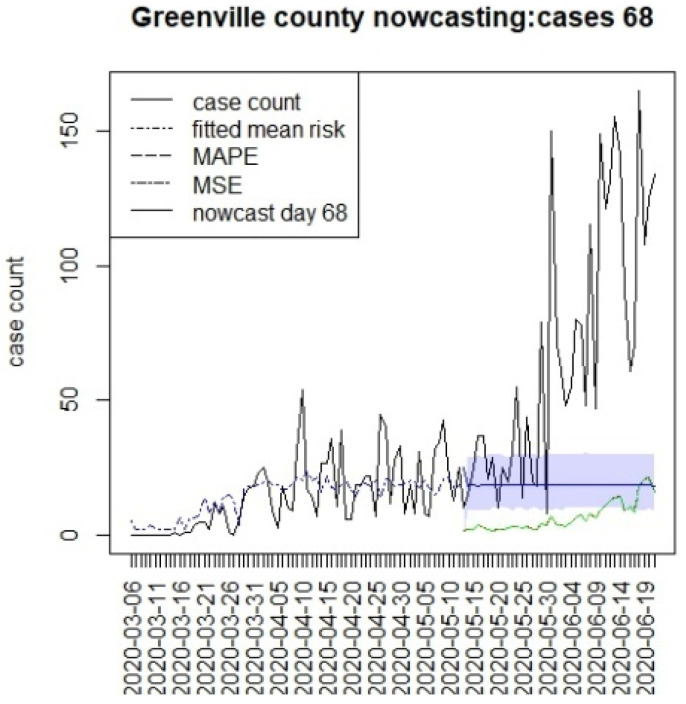
Counterfactual for Greenville county at T = 68.

**Figure 9:**
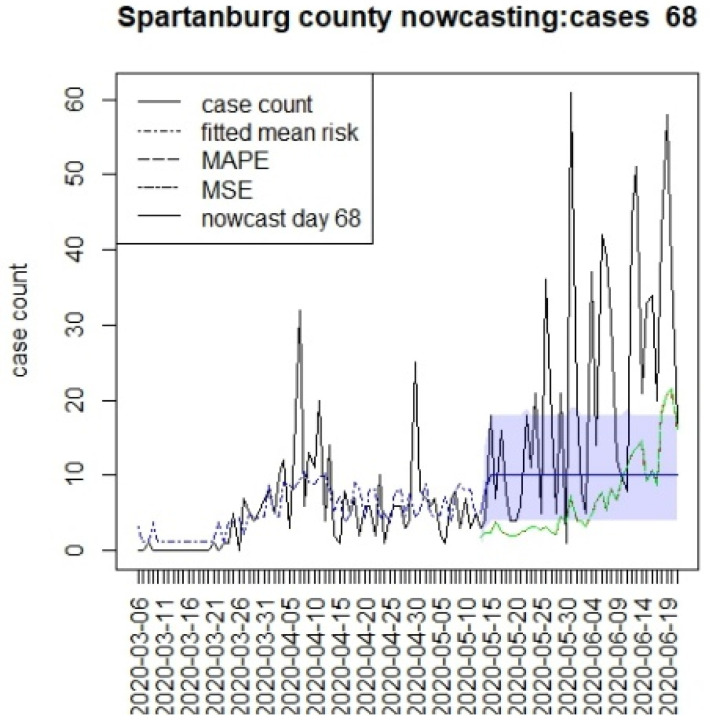
Counterfactual for Spartanburg county at T = 68.

Figure 6, Figure 7, Figure 8 and Figure 9 display the counterfactuals for the same four counties at time T = 68, which is the end of the lockdown period. We do not display the intermediate case time point here, nor the counterfactuals for deaths, for brevity.

In general, the figures show considerable between-county variation, but also a relatively close fit of the underlying model for a variety of counties. In contrast, the displays suggest differences between the counterfactuals and the observed counts, and it is more relevant to compute the summary measures of the differences. In Table 1, we present the results for estimating the mean differences between the counterfactuals and the observed counts. We define the difference at time k as

$$\begin{array}{l} e_{i,k}=y_{i,k}^{p}-y_{i,k}\;\mathrm{and}\;\mathrm{the}\;\mathrm{mean}\;\mathrm{difference}\;\mathrm{is}\;\underset{k}{mean}(e_{i,k}).\\ \mathrm{The}\;\mathrm{MAPE}\;\mathrm{is}\;\mathrm{given}\;\mathrm{by}\;(M)APE_{i,k}=abs(e_{i,k})\;\mathrm{and}\;\mathrm{the}\;\mathrm{MSE}\;\mathrm{by}\;(M)SE_{i,k}=e_{i,k}^{2}. \end{array}$$

These time-based loss measures are shown on the counterfactual figures. 

In Table 1, we display the average differential between the counterfactual and overserved count over the time period of prediction (16, 26, or 40). In this table, any negative difference in the overall mean levels represents a situation where the case load is higher than the predicted counterfactual.

This suggests that the in the first period, the prediction was everywhere lower than case counts, as there was limited lockdown. In the second period, Charleston and Richland achieved positive results as they remained under lockdown with lower case numbers, whereas Greenville remained negative. In fact, Greenville remained with a high case load throughout out the periods, and this suggested that compliance was poor in this county. However, Spartanburg had a similar pattern to Richland and Charleston. It is important to note that the early lockdowns did not help the case count in the second larger wave during the summer of 2020. All of the predictions returned negative mean differences during the final period. It is notable that the predictions across long lags tended to have wide credible intervals, and so, some degree of uncertainty in these estimates remains [15]. In addition, it is also notable that beyond the initial step predictions, the SIR model leads to almost constant overall risk mean levels. This is typically due to lack of future data support and the need for shocks within a SIR model to allow for peak generation.

## 8. New Jersey Counties 

We assumed that the crucial time points for this state, measured from 6 March, were T = {8,16,96}. The first marks the initial restrictions on 14 March, and the second, on 22 March, when a more restrictive lockdown was imposed. The last time for this was when the lockdown was finally lifted (10 June). In this case, we have examined a 40 day period beyond the T times to examine longer term lockdown effects. 

Figure 10, Figure 11, Figure 12, Figure 13, Figure 14, Figure 15, Figure 16, Figure 17, Figure 18, Figure 19, Figure 20 and Figure 21 display the results of fitting the model NJ1 and the posterior expected counterfactuals for the counties of Gloucester, Bergen, Hunterdon, and Middlesex. 

**Figure 10:**
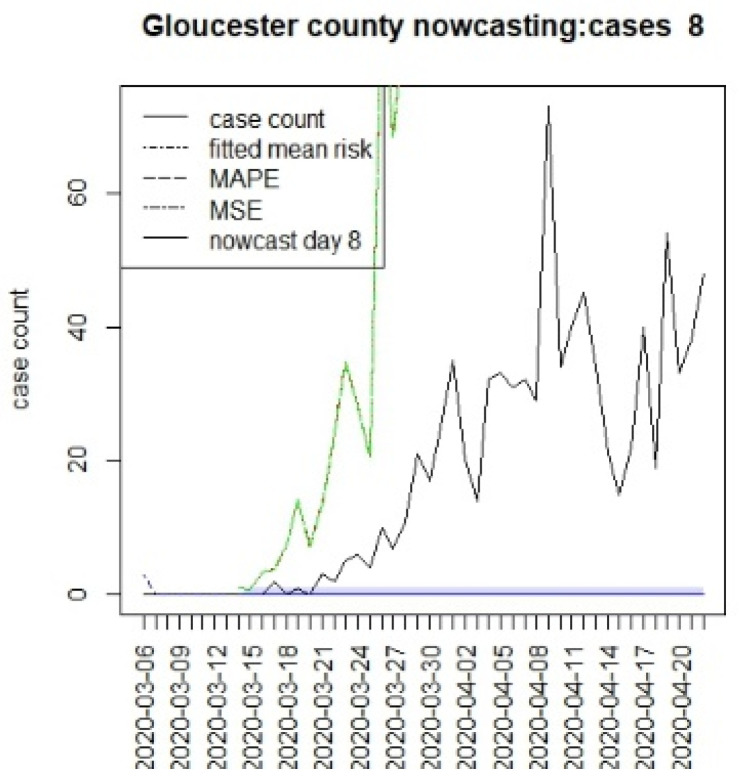
Counterfactual for Gloucester county, T = 8.

**Figure 11:**
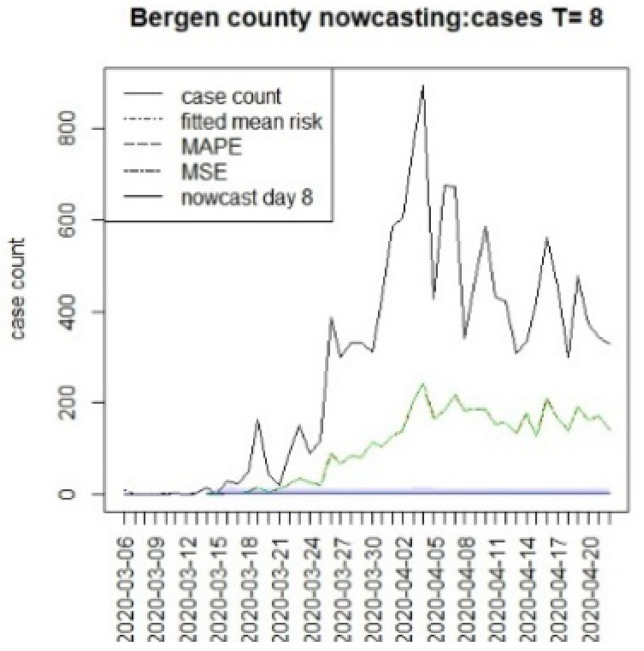
Counterfactual for Bergen county, T = 8.

**Figure 12:**
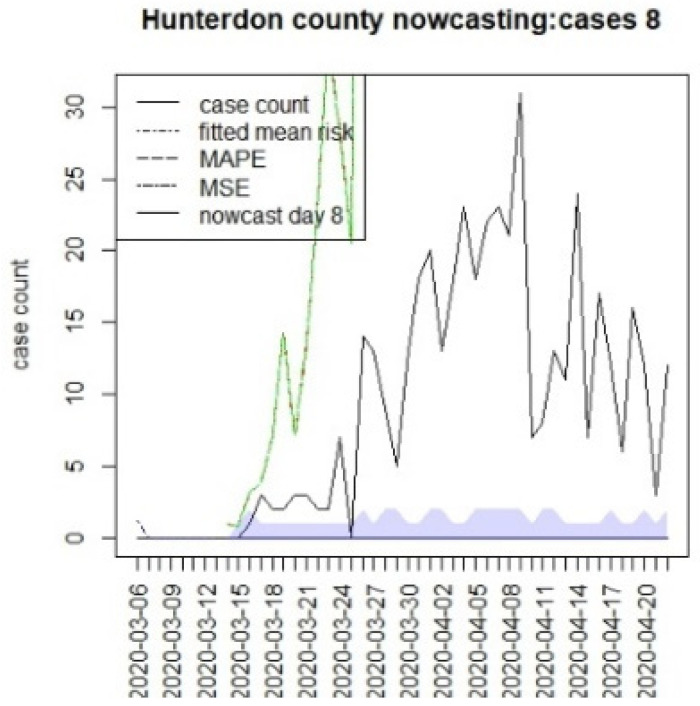
Counterfactual for Hunterdon county, T = 8.

**Figure 13:**
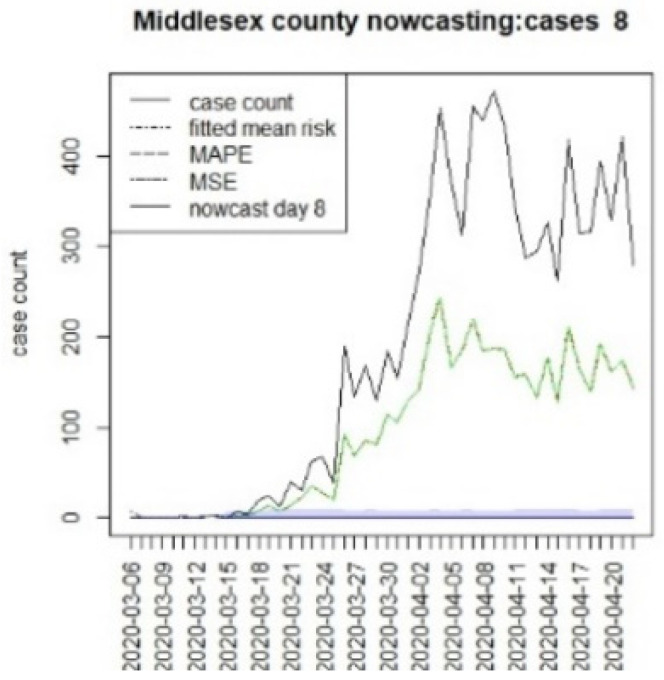
Counterfactual for Middlesex county, T = 8.

**Figure 14:**
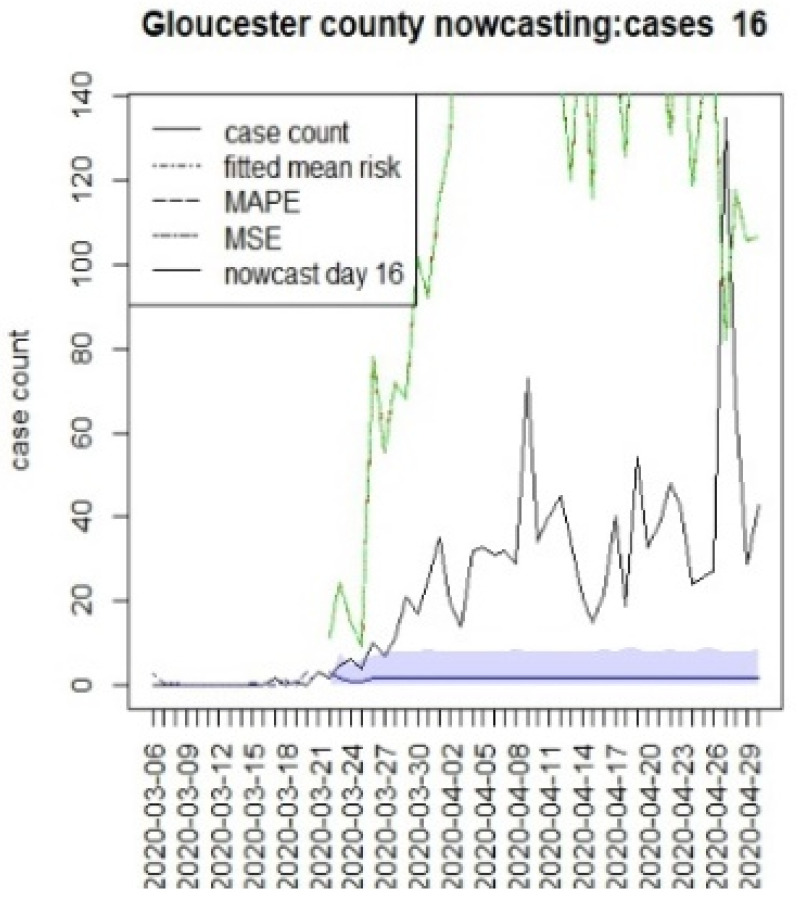
Counterfactual for Gloucester county, for T = 16.

**Figure 15:**
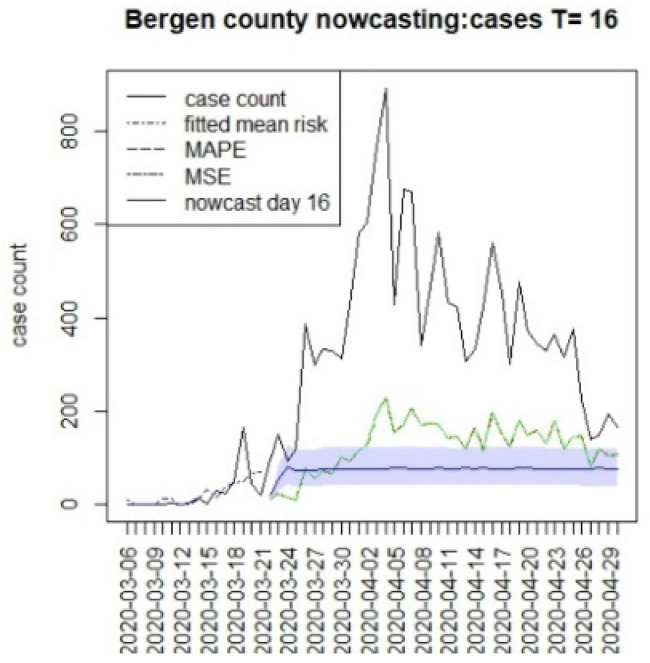
Counterfactual for Bergen county, T = 16.

**Figure 16:**
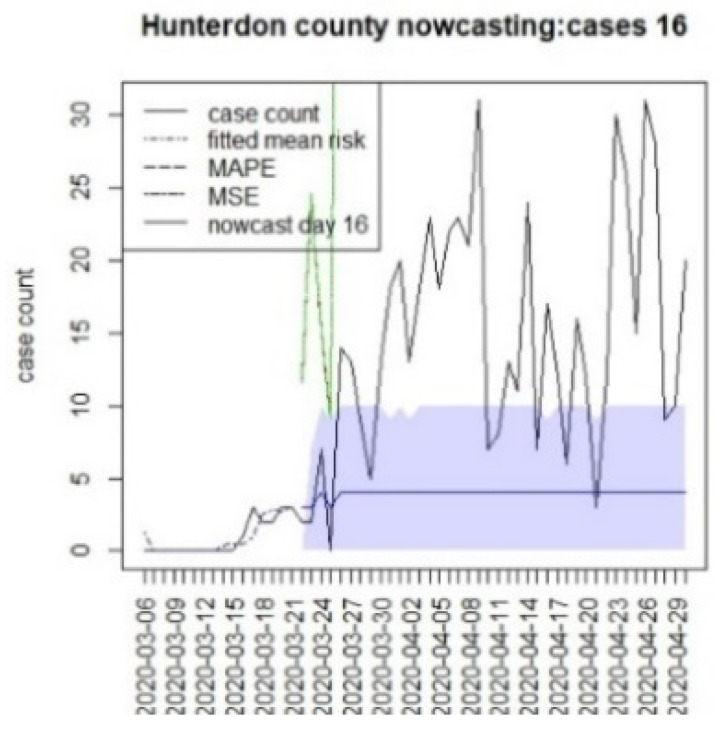
Counterfactual for Hunterdon county, T = 16.

**Figure 17:**
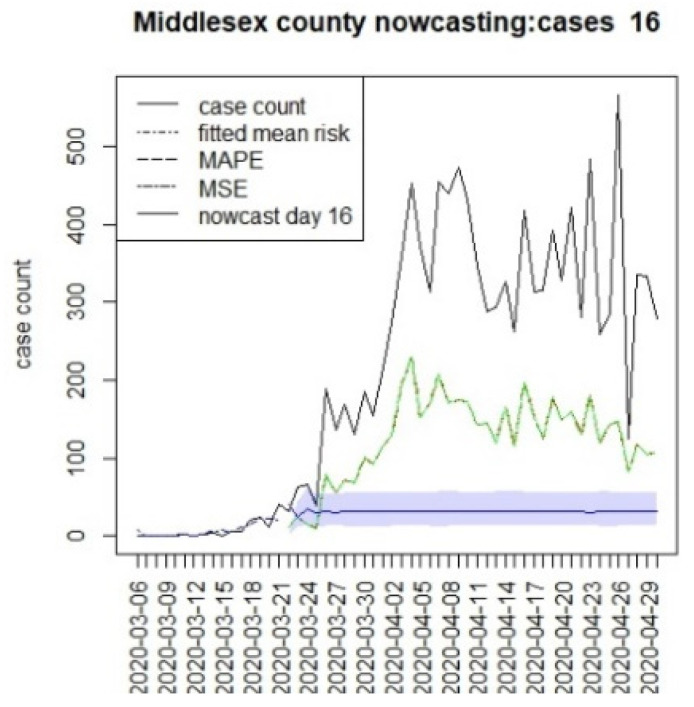
Counterfactual for Middlesex county, T = 16.

**Figure 18:**
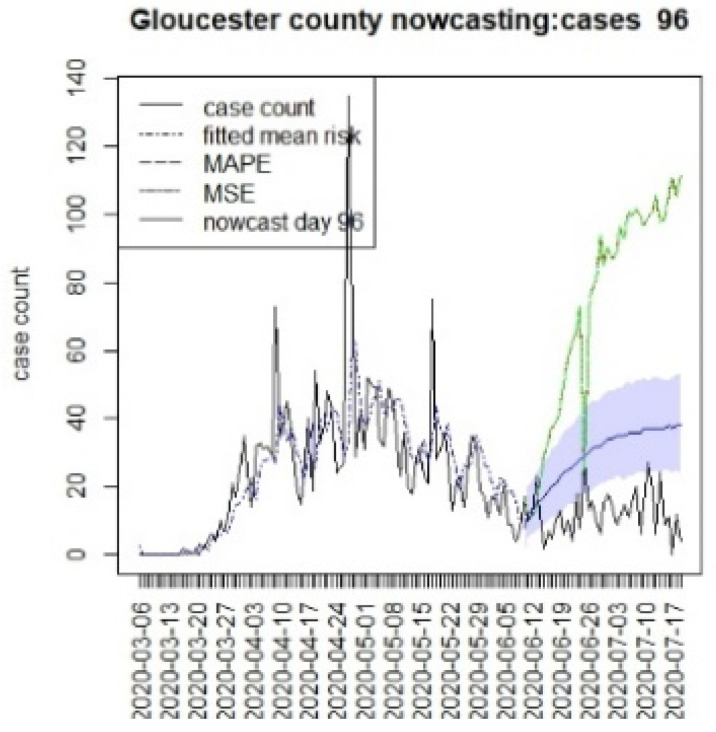
Counterfactual for Gloucester county, T = 96.

**Figure 19:**
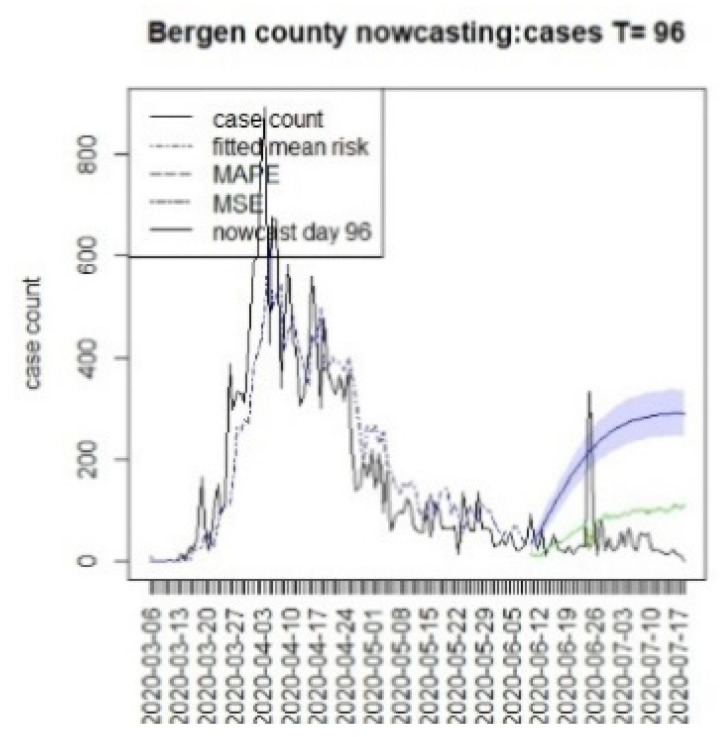
Counterfactual for Bergen county, T = 96.

**Figure 20:**
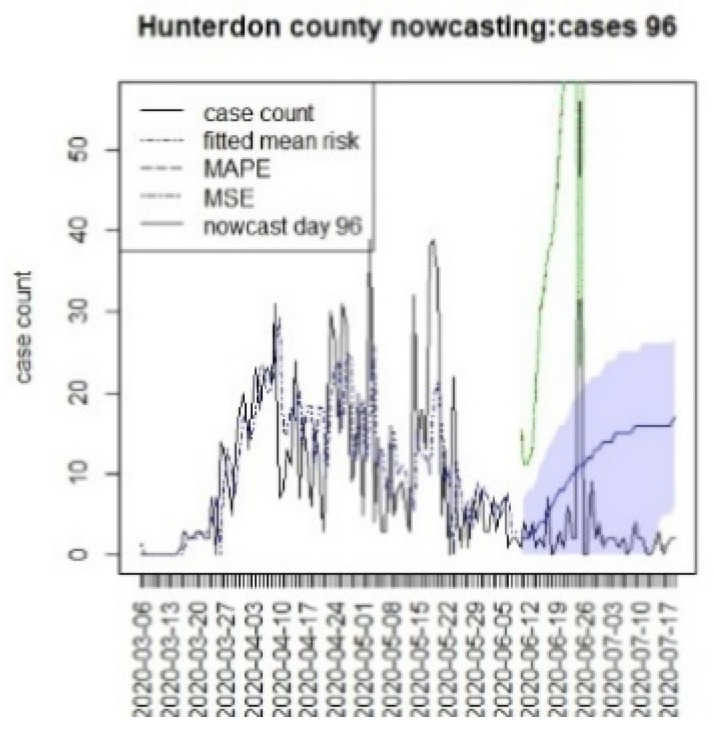
Counterfactual for Hunterdon county, T = 96.

**Figure 21:**
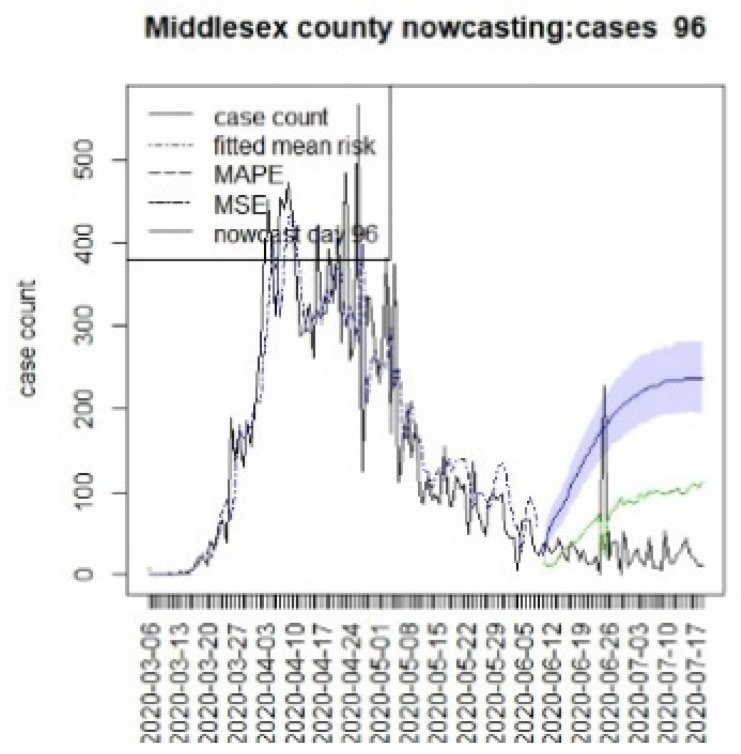
Counterfactual for Middlesex county, T = 96.

It is clear from Figure 10, Figure 11, Figure 12, Figure 13, Figure 14, Figure 15, Figure 16, Figure 17, Figure 18, Figure 19, Figure 20 and Figure 21 that a different pattern emerges for the nowcasting counterfactuals for NJ. At T = 8, the nowcasts under-report the observed case counts considerably. However, at T = 16, the situation improved with a higher rate of prediction, although it was still mainly below the observed case count. By T = 96, the observed cases were below the counterfactual. This is clearly reflected in Table 2, where the differentials become highly positive by T = 96. This suggests that at this point, the case load has been reduced significantly.

## 9. Discussion 

The counterfactual generation pursued in this paper has a number of drawbacks. First, long-term prediction has been shown to demonstrate very wide credible intervals (see e.g., Figure 2, Figure 3, Figure 4 and Figure 5). This means that the predictions are potentially variable and that they do not have high confidence. This would appear to be in part because of the SIR model form, but also as data support is limited, the further in the future the prediction is made. A second issue that arises with SIR model predictions is that the overall risk level becomes relatively constant over time. This is due to the lack of jumps in risk based on the final observed data point [15]. Although random effects are commonly used in Bayesian disease mapping as a way to deal with extra variation, there is a trade-off, as they might not be well estimated when the information fed in the system is too diffused, particularly for new emerging diseases. One of the major limitations of the analysis of these data is the issue of under-ascertainment, whereby biases appear in the count data. This could be due to reporting delays or unobserved infections (asymptomatics or under-reporting). In previous work, we have examined different biases and their effects [10]. The current models adjust for under-reporting via a rate. It is possible that this rate could vary over time, and so this is not catered for in this analysis. The existence of differential effects within strata in the population is also a major concern. It is possible that older age groups have a greater risk of infection or severity of outcome. Our data do not include a stratification of this kind, but it would be important in future applications that these strata effects are included when making public health decisions. Another aspect of this work is that well-fitting retrospective models were used to make predictions. This emphasizes time and space-averaged model responses. Shorter term predictive model fits (possibly based on lag windows) may be a future extension to be examined, along with data assimilation methods.

Nonetheless, this work has proposed an extension of the SIR Bayesian disease mapping framework to account for uncertainty in infectious surveillance. In addition, the range of prediction should be further examined to find the optimal predictive interval, since this could have an effect on both the accuracy and computing resources of surveillance activities in which timeliness is a key [17].

## 10. Conclusions

The approach proposed here highlights the differentials between both counterfactuals and observed (confirmed) case counts, as well as between regions and states.

With respect to the county differences, there is strong evidence for major differences in response to the interventions between counties in SC. Greenville county in particular shows continual case spread during the lockdowns. In other analyses, this the continued existence of the clusters of case counts in that county that support the conclusion that non-compliance was common there. The particular difference that is clear appears in the second period after T = 26, when Charleston and Richland had reduced case loadings, whereas Greenville remained above the counterfactual throughout the three periods.

In the case of New Jersey, the clear trend was for some success during the middle period, and then positive differentials after T = 96 across all counties, which suggests that suppression was achieved.

Furthermore, the comparison of states is marred by the fact that the lockdowns were of different kinds and durations. It is quite remarkable that the patterns of compliance are markedly different, both between states and within the states. SC did not succeed in locking down adequately, and it had no NPI in place for the second wave during the summer of 2020. In addition, a large difference remained between the counties within that state. Furthermore, New Jersey maintained their lockdowns and achieved a degree of suppression, with a similar pattern across counties. 

Finally, we note that the approach described here could have a sensitivity to the choice of T. However, the choice of T is usually defined by policy decisions, and so there is only a limited possibility to alter these times. The sensitivity to the choice of K could be apparent, but we believe that the due to the averaging effects across time spans, this is limited.

An advantage of this method is the fact that confidence intervals could be derived for differentials and functions of differentials of various kinds. Further advantages lie in the flexibility to specify different prior distributions for model parameters, and the ease with which the models can be fitted using standard R packages (Nimble). Here, we present basic averages that highlight differences between states and regions. In future work, we plan to refine our summarization of the differentials to better reflect the variability. In addition, we would examine the use of different data assimilation approaches to improve the predictions. A further future development would be to make a comparison of the current approach to the counterfactuals estimated from different types of prediction models. 

## Figures and Tables

**Table 1:** Mean differences between counterfactuals and observed counts averaged over the respective time periods. T is time point, and K is extent. Model assumed is SC1.

Time	Charleston	Richland	Greenville	Spartanburg
T26K16	−1.68	−11.1	−9.06	−1.37
T42K26	10.6	2.34	−4.00	6.57
T68K40	−23.8	−15.3	−48.6	−11.4

**Table 2:** Mean differences in counterfactuals and observed counts for four NJ counties based on the assumed best model, NJ1.

Time	Bergen	Gloucester	Hunterdon	Middlesex
T8K40	−338.6	−21.4	−10.9	−225.1
T16K40	−306.2	−29.2	−10.8	−258.8
T96K40	172.2	17.0	7.8	147.4

## Data Availability

Data are completely available from the NYT GitHub repository (https://github.com/nytimes/covid-19-data, accessed on 29 November 2022).
